# Molecular epidemiology of Crimean-Congo hemorrhagic fever virus in Russia

**DOI:** 10.1371/journal.pone.0266177

**Published:** 2022-05-12

**Authors:** Anna Volynkina, Yana Lisitskaya, Albert Kolosov, Lyudmila Shaposhnikova, Sergey Pisarenko, Vladimir Dedkov, Anna Dolgova, Alexander Platonov, Alexander Kulichenko

**Affiliations:** 1 Stavropol Research Antiplague Institute, Federal Service for Consumer Rights Protection and Human Well-being, Stavropol, Russia; 2 Saint Petersburg Pasteur Institute, Federal Service for Consumer Rights Protection and Human Well-being, Saint Petersburg, Russia; 3 Martsinovsky Institute of Medical Parasitology, Tropical and Vector Borne Diseases, Sechenov First Moscow State Medical University, Moscow, Russia; 4 Central Research Institute for Epidemiology, Federal Service for Consumer Rights Protection and Human Well-being, Moscow, Russia; CEA, FRANCE

## Abstract

In this report, we present new data on the diversity and geographical distribution of genetic variants in Crimean-Congo hemorrhagic fever virus (CCHFV) in Russia. Partial S, M, and L segment sequences of the CCHFV genome were obtained from 500 serum samples from CCHF patients and 103 pools of ticks collected in the south of the European region of Russia in 2007–2017. The investigated viral strains belonged to the lineages Europe 1 (596 samples), Africa 3 (1 sample) and a new genetic lineage, Europe 3 (6 samples). The Russian CCHFV strains of the Europe 1 lineage formed four subgroups (Va-Vd) correlated with the geographical site of virus isolation. Segment reassortment events between strains of different subgroups within lineage Europe 1 were revealed. The complete S, M and L genome segments of 18 CCHFV strains belonging to different subgroups of the Europe 1 lineage and the complete S segments of 3 strains of the Europe 3 lineage and 1 strain of the Africa 3 lineage were sequenced. The analysis of the geographical distribution of CCHFV genetic variants in southern Russia revealed local viral populations with partially overlapping boundaries.

## Introduction

Crimean-Congo hemorrhagic fever virus (CCHFV) is the etiological agent of Crimean-Congo hemorrhagic fever (CCHF), a severe disease in humans that can be transmitted via *Ixodidae* tick bites, contact with the tissues of infected animals, contact with the biological fluids of infected patients and vertical transmission [1]. It is endemic in the countries of Africa, Asia, and southeast Europe and in a number of regions in the south of the European region of Russia [2]. CCHFV belongs to the *Orthonairovirus* genus within the *Nairoviridae* family [3].

The epidemic situation of CCHF in Russia remains troublesome. In 1999–2020, 2,361 CCHF cases were detected in southern Russia, 94 of which (3.98%) had lethal outcomes. CCHF outbreaks were registered in ten administrative territories of Russia: Stavropol Territory (814 cases, 25 deaths in 1999–2020), the Rostov region (750 cases, 29 deaths in 1999–2020), the Astrakhan region (176 cases, 7 deaths in 1999–2020), the Volgograd region (158 cases, 11 deaths in 2000–2020), the Republic of Kalmykia (385 cases, 12 deaths in 2000–2020), the Republic of Dagestan (65 cases, 4 deaths in 2000–2020), the Republic of Ingushetia (6 cases, 5 deaths in 2004, 2007, and 2008), the Karachay-Cherkessia Republic (3 cases in 2007, 2008, and 2015), the Kabardino-Balkaria Republic (one fatal case in 2016), and the Republic of Crimea (1 case in 2017, and 2 CCHF cases were imported from Crimea to Moscow and Voronezh cases in 2013 and 2015). The gradual expansion of the territory of the natural focus of CCHF in southern Russia and the involvement of new regions in the epidemic have been noted.

CCHFV is one of the most genetically diverse arboviruses [4]. The virus genome consists of three segments of single-stranded RNA with negative polarity: the S segment (1,670 bp), M segment (5,360 bp), and L segment (12,160 bp). The S segment encodes the nucleoprotein (482 aa); the M segment encodes the GPC polyprotein (1,688 aa)–a protein precursor of the Gn and Gc surface glycoproteins; and the L segment encodes the RNA-dependent RNA polymerase (3,945 aa) [5,6].

Based on an analysis of complete and partial S segment sequences, seven genetic lineages of CCHFV were distinguished and shown to be correlated with the geographical site of virus isolation; these lineages were Africa 1, Africa 2, Africa 3, Asia 1, Asia 2, Europe 1, and Europe 2 [7,8]. A phylogenetic analysis of partial and complete M and L segment sequences revealed nine and six genetic lineages, respectively, that were partially congruent with the S segment-based lineages [1,8–10]. Segment reassortment and recombination, which occur in simultaneous infections of vectors with viral strains belonging to different lineages and lead to the formation of new genetic variants, are characteristic of CCHFV [11–13].

Previous studies have established that CCHFV strains of the Europe 1 lineage are circulating in southern Russia in Stavropol Territory and the Rostov, Volgograd, and Astrakhan regions [14–16]. Strains from Turkey, southeast Europe, and Iran also belong to this genetic lineage [4,17]. Genetic differences between CCHFV strains of lineage Europe 1 circulating in various geographical regions have been revealed. The phylogenetic analysis of partial S segment sequences allows several subgroups within lineage Europe 1 to be separated. Russian CCHFV strains belong to three subgroups: Stavropol-Rostov-Astrakhan (Va), Volgograd-Rostov-Stavropol (Vb), Astrakhan-2 (Vc), and Crimea (Vd) [16,18,19]. Strains from the Balkans and Turkey form distinct subgroups [20–22].

This study aimed to obtain new data on the genetic diversity of CCHFV in Russia, to reveal prevailing genetic variants and to compare their spatial distribution.

## Methods

### Serum and tick samples

The materials used in this study were serum samples collected from CCHF patients in Russia in 2007–2017 that were positive for the presence of CCHFV RNA and pools of ticks belonging to the species *Hyalomma marginatum*, *H*. *scupense*, *Rhipicephalus turanicus*, *Haemaphysalis punctata*, and *Dermacentor marginatus* collected in southern Russia (Table 1).

**Table 1:** Number of human serum samples and pools of ticks positive for the presence of CCHFV RNA used for genetic characterization of CCHFV isolates.

Region	Year	Sera of CCHF patients	Pool of *Ixodes* ticks
**Stavropol Territory**	2007–2016	264	21
**Krasnodar Territory**	2013–2016	-	3
**Rostov Region**	2011–2016	188	51
**Astrakhan Region**	2011–2016	12	9
**Volgograd Region**	2014–2016	12	-
**The Republic of Kalmykia**	2012–2016	21	13
**The Republic of Dagestan**	2013	1	
**Kabardino-Balkaria Republic**	2016	1	-
**The Republic of Crimea**	2017	1	6
**TOTAL**	2007–2017	500	103

Clinical samples of CCHF patients for whom site of infection was established were included in the study. The number of samples was correlated with the prevalence of CCHFV in each region. This study was approwed by the Stavropol Research Anti-Plague Institute’s ethics committee. All CCHF patients personal data were analyzed anonymously.

### Detection of viral RNA

Viral RNA was extracted from the clinical samples and tick suspensions using the RIBO-prep extraction kit (AmpliSens^®^, Russia), and complementary DNA was obtained using the REVERTA-L Kit (AmpliSens^®^, Russia). The detection of CCHFV in human sera and ticks was carried out using the RT-PCR CCHFV-FL Kit (AmpliSence^®^, Russia).

### Genotyping

CCHFV genotyping was performed by sequencing three fragments of the viral genome: a partial S segment (position 115–652), partial M segment (position 4620–5075), and partial L segment (position 105–541), which were then subjected to phylogenetic analysis. The genetic variants present in different CCHFV strains were determined through the comparison of the corresponding cluster positions in phylogenetic trees based on partial S, M and L segments.

Fragments of the S, M and L segments were amplified using the primer pairs S 100f and S 680r [18]; 24 and 25 [23]; and L100f: gattggactcaggtgattgctggtc and L540r: gcctccctcgtgtctgtttc, respectively. The purification of the PCR products was carried out using the AxyPrep™ PCR Cleanup Kit (Axygen Biosciences, USA). Amplicons were bidirectionally sequenced using a Big Dye Terminator Kit v. 3.1 on an ABI 3130 Genetic Analyzer (Applied Biosystems, USA).

### Complete genome sequencing

The complete S, M, and L segments of the CCHFV genome were amplified as 20 overlapping fragments using a set of primers (S1 Table). The concentration of the amplicons was assessed by performing electrophoretic separation in a 1% agarose gel. Equivalent amounts of each PCR product were combined, and the DNA was purified using the AxyPrep™ PCR Cleanup Kit (Axygen Biosciences, USA) and used to generate shotgun DNA libraries. The preparation of libraries with a mean read length of 200 bp and subsequent sequencing were performed using the Ion Xpress™ Plus Fragment Library Kit, Ion Xpress Barcode Adapters 1–16 Kit, Ion One Touch 200 Template Kit v2 DL, Ion PGM 200 Sequencing Kit, and Ion 314Chip Kit on an Ion Torrent Personal Genome Machine next-generation sequencer (Life Technology, USA).

### Phylogenetic analysis

Contigs were assembled using Newbler Assembler 2.9, Newbler Mapper 2.9 (454 Life Science), and Vector NTI 8.0 software. The nucleotide sequences of the partial and complete genome segments of CCHFV strains obtained in this study were compared with reference S, M and L segment sequences of CCHFV strains belonging to different genetic lineages available in GenBank (S2 Table). The phylogenetic analysis of partial S, M and L segment sequences was conducted via the neighbor-joining method according to the Kimura-2 algorithm using Mega 5.05 software. Phylogenetic trees based on complete S, M and L genome segments were generated via the neighbor-joining method in Mega 5.05 software (Kimura-2 algorithm, statistical significance of the phylogenetic tree topology was assessed in the bootstrap test with 1000 replications) [24] and by using BEAST 2.0 (GTR+G model, strict clock, 10,000,000 generations) [25]. The MCC tree files were visualized using FigureTree 1.4.3

### Geographic distribution analysis

The geographical distribution of genetic variants of CCHFV was analyzed using ArcGIS 10.1 software.

## Results

### Genetic diversity of CCHFV based on partial genome segments

Partial S, M, and L segments of the CCHFV genome sequences were obtained from 500 serum samples of CCHF patients and 103 pools of ticks collected in Stavropol Territory, the Rostov Region, Astrakhan Region, Volgograd Region, Republic of Dagestan, Republic of Kalmykia, Kabardino-Balkarian Republic and Krasnodar Territory. Partial sequences were submitted to the GenBank database (S1 File).

**Fig 1:**
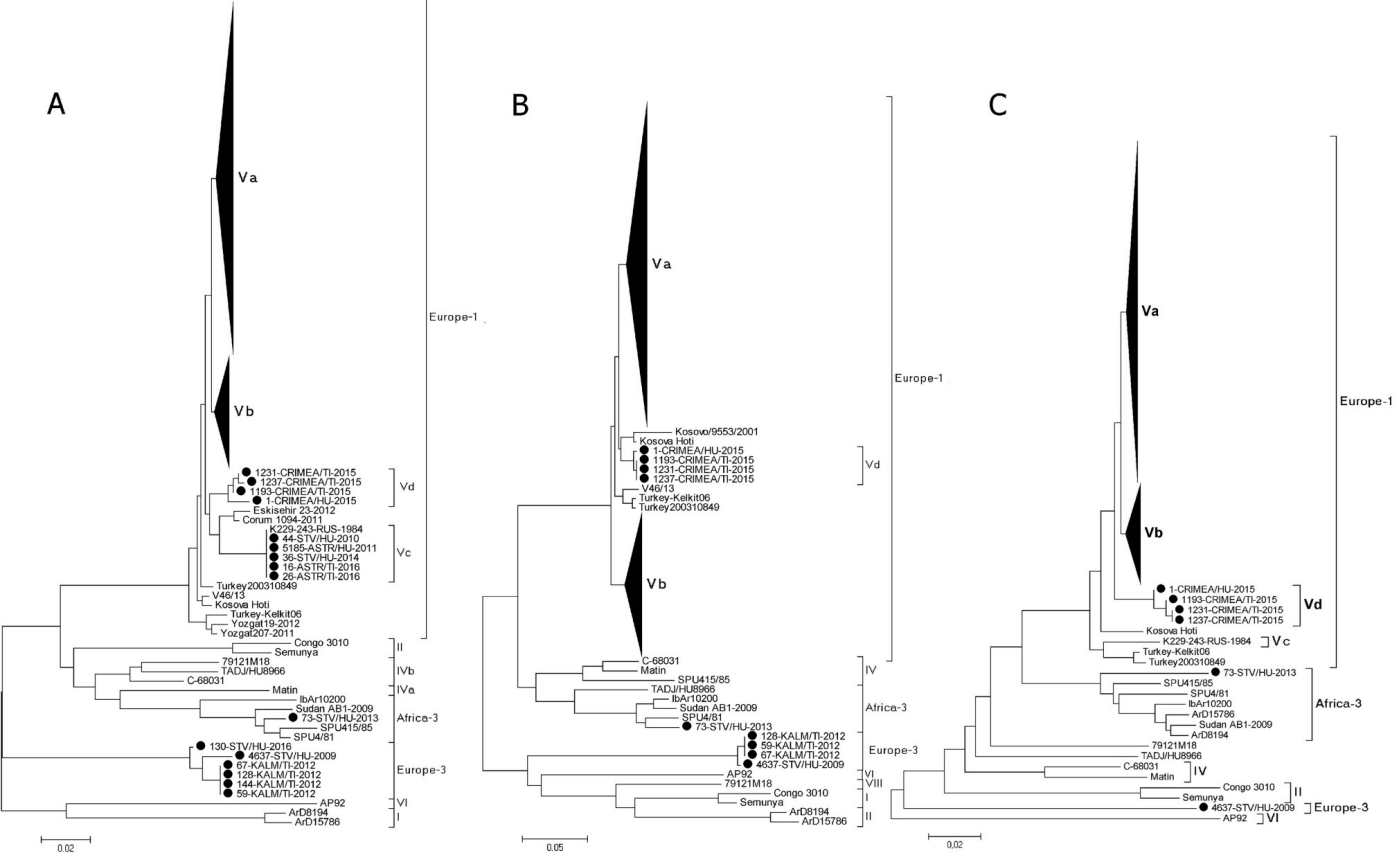
Neighbor-joining phylogenetic trees based on: A: A 538 bp fragment of the S segment; B: A 435 bp fragment of the M segment; C: A 437 bp fragment of L segment; sequences from the present study are marked.

The phylogenetic trees constructed on the basis of the partial S, M and L segment sequences obtained in the present study and reference genome sequences of CCHFV strains available in the GenBank database are presented in Fig 1. The phylogenetic analysis of the partial segment sequences showed that 596 of the investigated CCHFV strains belonged to genetic lineage Europe 1 (497 strains from human sera and 99 strains from ticks of the species *H*. *marginatum*, *H*. *scupense*, *R*. *turanicus*, *D*. *marginatus* and *H*. *punctata)*. One strain (73-STV/HU-2013) isolated from the serum of CCHF patients in Stavropol Territory collected in 2013, belonged to lineage Africa 3 (III). The minimal percentage of nucleotide differences between isolate 73-STV/HU-2013 and the other strains of the Africa 3 lineage was 2% for the partial S segment, 4% for the partial M segment and 5% for the partial L segment.

The CCHFV strains isolated from two serum samples from patients from Stavropol Territory collected in 2009 and in 2016 (4637-STV/HU-2009 and 130-STV/HU-2016) and four pools of *H*. *marginatum* ticks collected in 2012 in the Republic of Kalmykia (59-KALM/TI-2012, 67-KALM/TI-2012, 128-KALM/TI-2012, and 144-KALM/TI-2012) formed a new branch, designated Europe 3, in the phylogenetic trees based on the partial S M and L segments. The percentage of nucleotide differences between the strains of the Europe 3 lineage and the strains belonging to other lineages ranged from 13–15% for the S segment 24–25% for the M segment and 16,7–20,1% for the L segment. The phylogenetic trees based on the partial S and L segments confirmed the division of the Russian CCHFV strains into four subgroups within lineage Europe 1: a Stavropol-Rostov-Astrakhan (Va) strain, a Volgograd-Rostov-Stavropol (Vb) strain, an Astrakhan-2 (Vc) strain and a Crimea (Vd) strain (Fig 1A and 1C). The Balkan and Turkish strains were also separated into isolated groups. In the M segment tree, the Russian strains belonged to only three subgroups within the Europe 1 lineage: Va, Vb and Vd (Fig 1B). Subgroup Vc was not distinguished in the M segment phylogenetic tree and consisted of only one strain (К229–243, isolated in the Astrakhan region in 1984) in the L segment tree. The investigated CCHFV strains that formed subgroup Vc in the S segment phylogenetic tree belonged to subgroups Va (44-STV/HU-2010 and 36-STV/HU-2014) and Vb (5185-ASTR/HU-2011, 16-ASTR/TI-2016, 26-ASTR/TI-2016) in M segment tree and to subgroup Va in the partial L segment tree.

The comparison of the topology of the S, M and L segment phylogenetic trees showed that the majority of the investigated CCHFV strains clustered in the same genetic subgroup and belonged to the following genetic variants: Va-Va-Va (408 samples), Vb-Vb-Vb (131 samples), and III-III-III–Africa-3 (1 sample). In addition, there were a number of CCHFV strains, probably reassortant, whose partial S, M and L segment sequences clustered in different subgroups within genetic lineage Europe 1, belonging to the following genetic variants: Va-Vb-Va (40 samples), Vb-Va-Va (5 samples), Vc-Vb-Va (3 samples), Vc-Va-Va (2 samples), Va-Vb-Vb (2 samples), Vb-Vb-Va (2 samples), and Vb-Va-Vb (1 sample).

### Genetic diversity of CCHFV based on complete genome segments

The whole-genome sequencing of 18 CCHFV strains representing different genetic variants within the Europe 1 genetic lineage, along with the complete S segments of three strains of genetic lineage Europe 3 and one strain of genotype Africa 3, was performed. the full-length nucleotide sequences were submitted to GenBank and are included in the S1 File.

**Fig 2:**
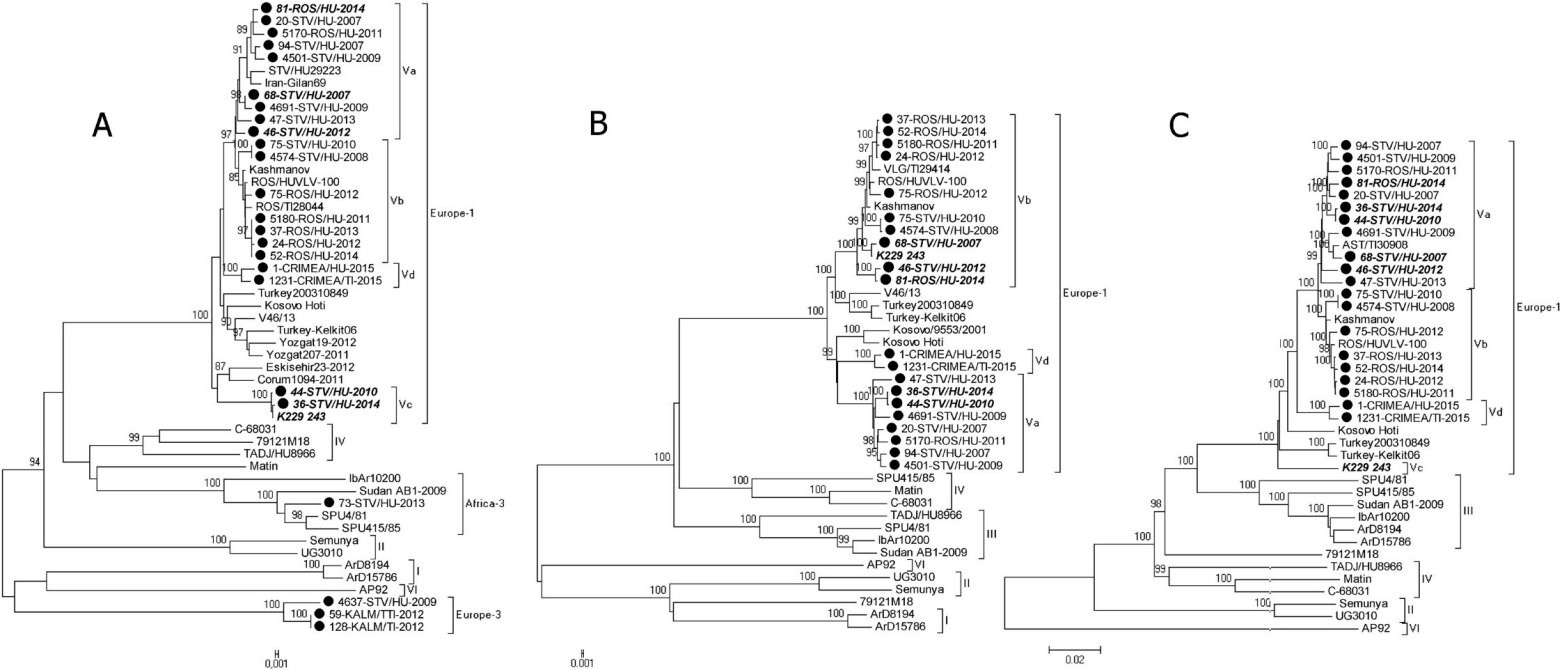
Neighbor-joining phylogenetic trees based on: A: The complete ORF of the S segment; B: The complete ORF of the M segment; C: The complete ORF of the L segment. The bootstrap test (1,000 replicates) results are shown next to the branches. sequences from the present study are marked. Reassortant isolates are indicated in bold.

The obtained complete sequences of the S, M and L segments of Russian CCHFV strains were used for phylogenetic analysis. The phylogenetic analysis of the complete coding regions (ORFs) of the S, M and L segments was conducted by neighbor joining (Fig 2) and Bayesian methods (S2 File). The topology of the phylogenetic trees based on the partial S, M and L segment sequences and the complete segment sequences and constructed by different methods were similar. The phylogenetic analysis of the complete segment sequences confirmed the formation of the distinct Europe 3 genetic lineage and the separation of subgroups Va-Vd within the Europe 1 lineage (NJ bootstrap support values for the subgroups, 70–100; posterior probability, 0.97–1). Reassortment events were confirmed for strains 81-ROS/HU-2014, 68-STV/HU-2007, 46-STV/HU-2012, 44-STV/HU-2010, 36-STV/HU-2014 and K229-243.

### Territorial distribution of CCHFV genetic variants in Russia

The geographical distribution of the genetic variants of CCHFV in southern Russia was analyzed. The coordinates of the tick collection points and the infection sites of the CCHF patients established during the epidemiological analysis of cases were plotted on a map of the southern portion of the European region of Russia (S1 File).

**Fig 3:**
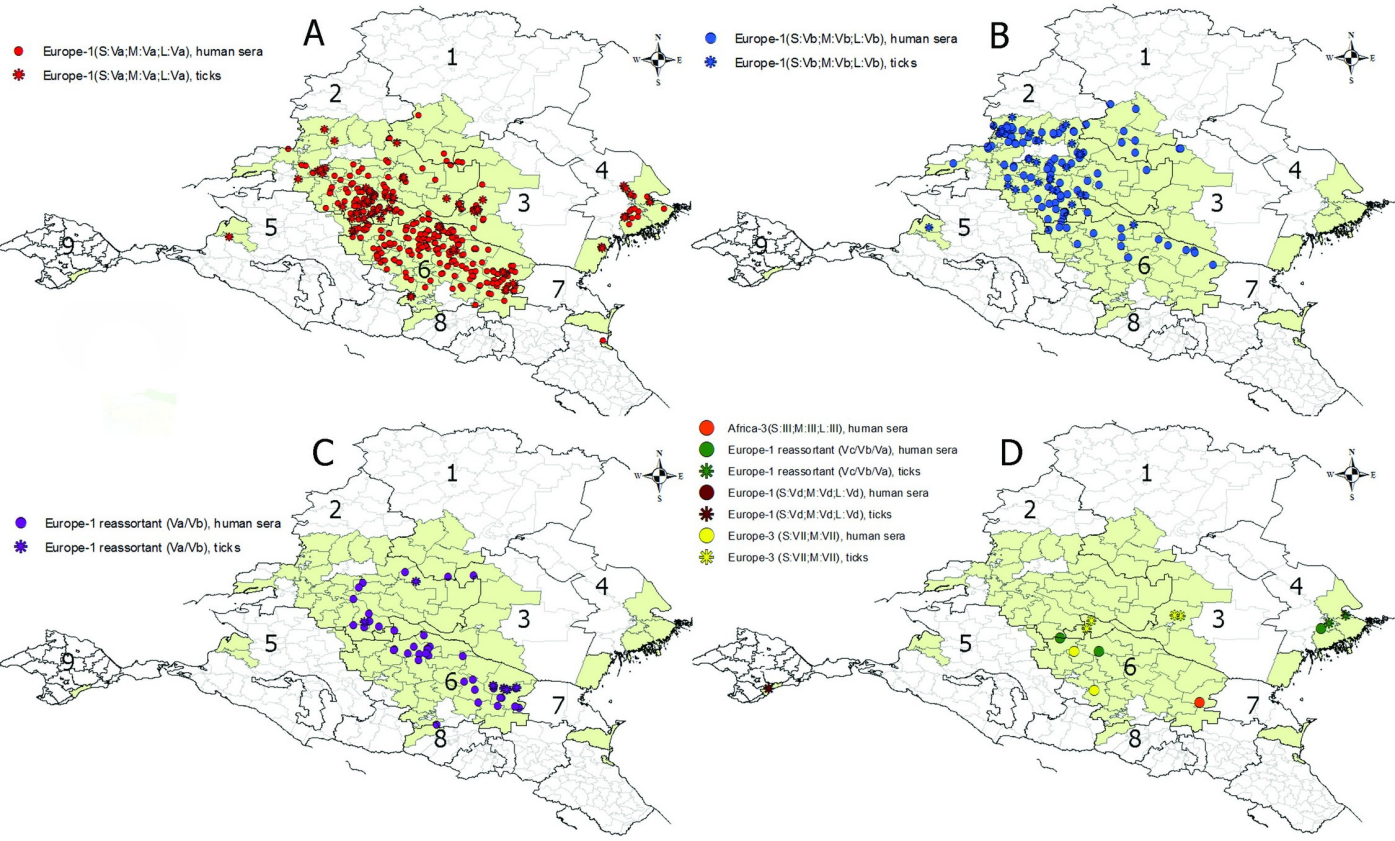
Geographic distribution of genetic variants of CCHFV in Russia. A: Europe 1, subtype Va, B: Europe 1, subtype Vb, C: Europe 1, reassortant variants between subtypes Va and Vb, D: Europe 1, subtype Vd, Europe 1, reassortant variants between subtypes Va, Vb and Vc, Africa 3, Europe 3. 1: Volgograd region, 2: Rostov region, 3: Republic of Kalmykia; 4: Astrakhan region, 5: Kransnodar territory, 6: Stavropol territory, 7: Republic of Dagestan, 8- Kabardino-Balkaria Republic; 9- Republic of Crimea. The districts where CCHF patient sera and ticks were collected are indicated in green. Esri reserves the right to grant permission for any other use of the Image.

The analysis of the geographical distribution of the genetic variants of CCHFV in southern Russia showed the presence of local viral populations with partially overlapping boundaries. CCHFV strains belonging to the Va-Va-Va genetic variant are circulating in the southern part of the CCHF natural focus and the Vb-Vb-Vb virus strains in the northern part (Fig 3A and 3B). Reassortant variants of CCHFV most often occur in the region of overlapping distribution areas of genetic variants Va-Va-Va and Vb-Vb-Vb (Fig 3C). Reassortant Vc-Vb-Va variants were revealed only in the Astrakhan region, and two isolates of the Vc-Va-Va genetic variant were found in the samples from Stavropol Territory. CCHFV strains belonging to subgroup Vd were detected only on Crimean Peninsula [19], and strains of the Europe 3 genetic lineage were detected only in the territory of the Republic of Kalmykia and Stavropol Territory. A single CCHFV strain of lineage Africa 3 was revealed east of Stavropol Territory (Fig 3D).

The prevalence of the circulating genetic variants of CCHFV in the administrative areas of the southern European part of Russia is presented in S1 Fig.

### CCHF severity during infection by different CCHF virus genovariants in Russia

Clinical data (severity rate, the presence of hemorrhagic manifestations and clinical outcome) of patients infected with different genovariant of CCHF virus were analysed (Table 2).

**Table 2:** Comparison of CCHF severity in Russia depending on the virus variant.

Genetic lineage	Desiase severity			Hemorrhagic syndrome		Clinical outcomes		Number of cases (abs.)
	Mild (%)	Moderate (%)	Severe (%)	No (%)	Yes (%)	Non fatal (%)	Fatal (%)	
**Europe-1**	1,4	82,5	15,8	72,1	27,9	97,6	2,2	498
**Va-Va-Va genetic variant**	2,0	83,9	14,0	72,8	27,2	98,2	1,5	342
**Vb-Vb-Vb genetic variant**	0,0	79,1	20,9	69,1	30,9	95,5	4,5	110
**Vd-Vd-Vd genetic variant**	0,0	0,0	100,0	0,0	100,0	100,0	0,0	1
**Va-Vb-Va genetic variant**	0,0	78,8	21,2	75,8	24,3	97,0	3,0	33
**Other reassortment variants**	0,0	91,7	8,3	75,0	25,0	100,0	0,0	12
**Europe-3**	0,0	100,0	0,0	100,0	0,0	100,0	0,0	2
**Africa-3**	0,0	100,0	0,0	100,0	0,0	100,0	0,0	1

The results showed risk severe clinical manifestation, hemorrhagic syndrome and fatal outcome increase for CCHFV by Europe-1 lineage (genetic variants Va-Vb-Va and Vb-Vb-Vb) compare with lineages Europe-3 and Africa-3. The small number of CCHF cases caused by CCHF virus, belonged to Europe-3 and Africa-3 lineages and some variants within genetic lineage Europe 1 is a limitation of the study.

## Discussion

In the southern European part of Russia, the strains of lineage Europe 1 dominate the CCHFV population, and strains of this genotype are typical for this region. The Russian CCHFV strains of the Europe 1 genotype form four subgroups, correlated with the geographical sites of sample isolation. Previously it was shown that isolates from distinct regions of South Russia were intermixed [26], however analysis of geographical distribution of the genetic variants of CCHFV in southern Russia based on coordinates of virus isolates collection points confirmed the presence of local viral populations with partially overlapping boundaries. The evidence of segment reassortment between strains of different subgroups within lineage Europe 1 were revealed based on partial and complete genome sequences. Notably, all CCHFV strains belonging to subgroup Vc in the S segment phylogenetic tree, which were described in this work and are available in the GenBank database, were reassortant. Rarely, divergent isolates of Europe-1 were also found in Russia [26]. The separation of strains isolated from various regions of Russia, Europe and Turkey into different subgroups within the Europe 1 genetic lineage provides evidence of the independent evolution of CCHFV genetic variants circulating in local CCHFV populations formed in geographically remote and isolated areas. Due to the subsequent migration of infected ticks (the main vectors of CCHFV) on birds, the genetic variants of CCHFV gradually spread into new areas [27]. The circulation of several genetic variants in a region may lead to the formation of reassortant strains of CCHFV.

Isolates of the Africa 3 genotype and of the new genetic lineage Europe 3 were revealed for the first time in the studied region.

The identification of a CCHFV isolate belonging to the Africa 3 genotype in a serum sample from an CCHF patient from southern Russia (the patient did not leave the Stavropol territory and was bitten by ticks while gardening at home) confirms the possibility of the long-distance transport of CCHFV in infected ticks on migratory birds or livestock. However, no CCHFV strain of lineage Africa 3 has yet been detected in ticks collected from birds or livestock in Russia.

The detection of strains of the new genetic lineage Europe 3 provides new data on the genetic diversity of CCHFV in Russia and the world. The percentage of the detected strains belonging to lineage Europe 3 among the studied strains was extremely low (1%). Two cases of CCHF caused by virus, belonged to lineage Europe-3 were moderate severity, without hemorrhagical manifestation, with non-fatal outcome. Most likely, the Europe 3 lineage is a more ancient genetic lineage of CCHFV than the Europe 1 lineage. Strains of this lineage were first introduced into the territory of Russia and circulated in natural foci until they were replaced by strains of lineage Europe 1, which now predominates and causes 99.9% of CCHF cases recorded in Russia. Further studies of CCHFV strains belonging to this genetic lineage, including the evaluation of the geographical distribution of strains of this lineage in Russia and other countries and the sequencing of the complete M and L segments, are required. This will make it possible to estimate the genetic diversity and spatial distribution of the Europe 3 genetic lineage.

## Supporting information

S1 Fig The prevalence of genetic variants of CCHFV in the districts in southern Russia. 1: Volgograd region, 2: Rostov region, 3: Republic of Kalmykia; 4: Astrakhan region, 5: Kransnodar territory, 6: Stavropol territory, 7: Republic of Dagestan, 8: Kabardino-Balkaria Republic, 9: Republic of Crimea. The districts where the investigated samples of CCHF patient sera and ticks were collected are indicated in green.
(TIF)

S1 Table Primer set for the amplification of the complete S, M, and L segments of the CCHFV genome. (DOC)

S2 Table Reference nucleotide sequences of CCHFV retrieved from GenBank used in the study. (DOC)

S1 File Partial and complete genome sequences of Russian CCHFV RNA isolates obtained in the study. (XLSX)

S2 File MCC tree, based on the complete ORF of the S segment. The ages of the clades are shown at the node. Reassortant strains are marked.
(DOC)
