# Supplementary figures and images for: Molecular epidemiology of Crimean-Congo hemorrhagic fever virus in Russia
Source: PLoS One. 2022 May 12;17(5):e0266177. doi: 10.1371/journal.pone.0266177 (PMC9098019; doi:10.1371/journal.pone.0266177)

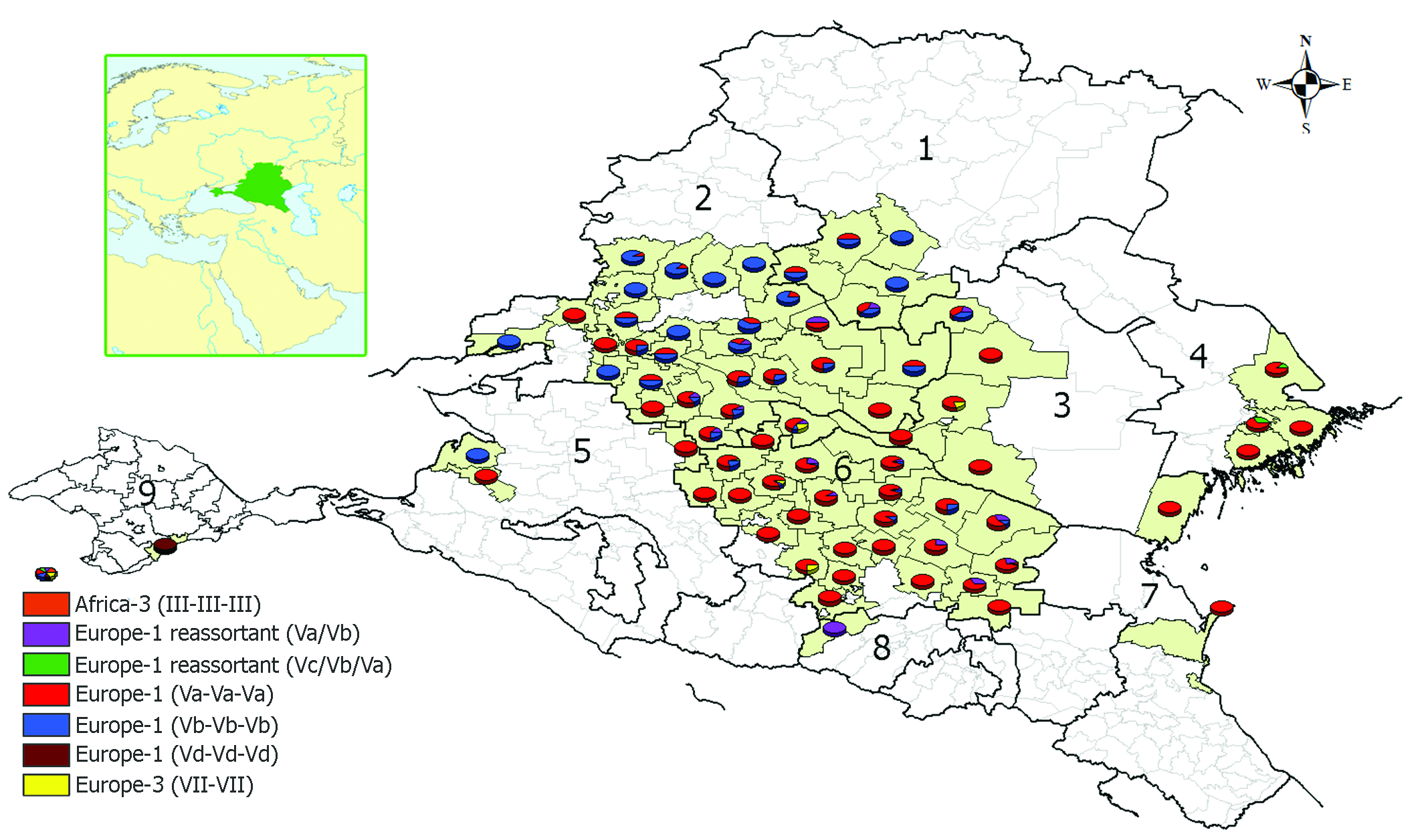

Supplement: S1 Fig — 1: Volgograd region, 2: Rostov region, 3: Republic of Kalmykia; 4: Astrakhan region, 5: Kransnodar territory, 6: Stavropol territory, 7: Republic of Dagestan, 8: Kabardino-Balkaria Republic, 9: Republic of Crimea. The districts where the investigated samples of CCHF patient sera and ticks were collected are indicated in green. (TIF) [file pone.0266177.s001.tif]
