# Supplementary material for: Molecular epidemiology of Crimean-Congo hemorrhagic fever virus in Russia
Source: PLoS One. 2022 May 12;17(5):e0266177. doi: 10.1371/journal.pone.0266177 (PMC9098019; doi:10.1371/journal.pone.0266177)
Supplement: S1 Table — (DOC) [file pone.0266177.s002.doc]

**Supplementary Table 1**

**Primer set for amplification of the complete S, M, and L segments of the CCHFV genome**

| **№** | **Locus**  **(amplicon size, bp)** | **Primer** | **5'-3' sequences** | **References** |
| --- | --- | --- | --- | --- |
| 1 | S_1-1490  (1490 bp) | CCHF-SF | TCTCAAAGAAACAAGTGCCGC | [Deyde et al, 2006] |
|  |  | S-680R | CACTGGTGGCATTGCCCTTGA | [Volynkina et al, 2012] |
| 2 | S_566-1673  (1108 bp) | S-580F | TCAGACATGATTAGRAGGAGGAA | [Volynkina et al, 2012] |
|  |  | CCHF-SR | TCTCAAAGATATCGTTGCCGC | [Deyde et al, 2006] |
| 3 | M_1-966  (966 bp) | CCHF-MF | TCTCAAAGAAATACTTGCGGCA | [Deyde et al, 2006] |
|  |  | MS1R | ACCCAAGGTYTCTCTTRCACCAYTC | This work |
| 4 | M_799-1752  (954 bp) | MS2F | TACATCCMAGCCCAACRAAYAG | This work |
|  |  | MS2R | TAGTTATTTTRTCACCYGGMCCACC | This work |
| 5 | M_1613-2523  (911 bp) | Gn1F | AGGCAAGGCATCAACWGGYT | This work |
|  |  | Gn2R | ACTCTGACACTTGCAAYAGCTTYCT | This work |
| 6 | M_2325-3306  (982 bp) | MS_NSF | GCAATAGAYGCTGARATGCAYGA | This work |
|  |  | MS_NSR | CATTTATTGCYCCCCAYGGTGCSTC | This work |
| 7 | M_3173-4486  (1314 bp) | Gc1F | CTGTCCATAYGAAGCTCTWGTRCT | This work |
|  |  | Gc3R | ACTTTRCAGGAGATRCCAGARCT | This work |
| 8 | M_4370-5364  (995 bp) | Gc4F | GGTTGARGTTGCTGACATGGAGT | This work |
|  |  | CCHF-MR | TCTCAAAGATATAGTGGCGGCA | [Deyde et al, 2006] |
| 9 | L_1-1496  (1496 bp) | CCHF-L1F | TCTCAAAGATATCAATCCCCCC | [Deyde et al, 2006] |
|  |  | LS1R | TGAGCCGAGAAGTTAATTGRTTGAG | This work |
| 10 | L_1011-2473  (1463 bp) | LS2F | GTGGGCTGTTGAGGGCWGC | This work |
|  |  | LS2R | CTCCTCAACTATTCCYCTYTCTC | This work |
| 11 | L_2202-3452  (1251 bp) | LS3F | TCTCCATTGATGTAACRCTGCCTGA | This work |
|  |  | LS3R | TAAGGTATTCCACATTRCGYTTGA | This work |
| 12 | L_3165-4458  (1294 bp) | LS4F | AGAAAACCGTTGAYGARACTGCYAA | This work |
|  |  | LS4R | TCAAACCTACCCTCAAGWGCAAARG | This work |
| 13 | L_4240-5480  (1241 bp) | LS5F | GCAAGGACCATTCTATYTGAAYAG | This work |
|  |  | LS5R | TCGGGTCYCTCCTTGCYTTRTCTAT | This work |
| 14 | L_5052-6321  (1270 bp) | LS6F | TAGGTTGCCCAAATGTKAGRAAAGC | This work |
|  |  | LS6R | TTGTAGTGTAAGCACCARGGRGCTG | This work |
| 15 | L_6067-7454  (1388 bp) | LS7F | AGATTTCGCAARCATCAATGAYA | This work |
|  |  | LS7R | TTAAYGCCATGAGTCCYTTRCTTAT | This work |
| 16 | L_7174-8498  (1352 bp) | LS8F | CCCAATACACTGCTGTTCYTTYTT | This work |
|  |  | LS8R | TCTTACCATCTCTYAGCCAATCRC | This work |
| 17 | L_8033-9497  (1465 bp) | LS9F | ACTTTGTCTCCCTTTGGYAGGYTGTT | This work |
|  |  | LS9R | TGAACACCGAGGGGAARAAYTGCAT | This work |
| 18 | L_9225-10491  (1267 bp) | LS10F | TAAACCTTCARTTCTCCGAYCCRAA | This work |
|  |  | LS10R | GGCTCGCTYTCAGCATCAAAYAC | This work |
| 19 | L_10041-11499  (1459 bp) | LS11F | CCACTGAACTGTTGAAGAARAAGCC | This work |
|  |  | LS11R | GCATGATTTAATGACCTYCTRGC | This work |
| 20 | L_11199-12157  (959 bp) | LS12F | TCAACCAGTGYAAGGCAGTAAARGA | This work |
|  |  | CCHF-L2R | TCTCAAAGAAATCGTTCCCCCCA | [Deyde et al, 2006] |
