# Supplementary material for: Molecular epidemiology of Crimean-Congo hemorrhagic fever virus in Russia
Source: PLoS One. 2022 May 12;17(5):e0266177. doi: 10.1371/journal.pone.0266177 (PMC9098019; doi:10.1371/journal.pone.0266177)
Supplement: S2 Table — (DOC) [file pone.0266177.s003.doc]

**Supplementary Table 2**

**Reference nucleotide sequences of CCHFV retrieved from the GenBank database used in the study**

| **Strain** | **Country** | **Year** | **Genetic lineage** | **GenBank Accession nos.** | | |
| --- | --- | --- | --- | --- | --- | --- |
|  |  |  |  | **S-segment** | **M-segment** | **L-segment** |
| ArD8194 | Senegal | 1969 | Africa-1 | DQ211639 | DQ211626 | DQ211613 |
| ArD15786 | Senegal | 1972 | Africa-1 | DQ211640 | DQ211627 | DQ211614 |
| Semunia | Uganda | 1958 | Africa-2 | DQ076413 | DQ094832 | DQ076412 |
| Congo3010 | Congo Repulic | 1956 | Africa-2 | DQ144418 | DQ019222 | DQ099335 |
| IbAr10200 | Nigeria | 1966 | Africa-3 | CHU88410 | CHU39455 | NC005301 |
| SPU4/81 | Republic of South Africa | 1981 | Africa-3 | DQ076416 | DQ157175 | DQ076417 |
| SPU415/85 | Republic of South Africa | 1985 | Africa-3 | DQ211648 | DQ211635 | DQ211622 |
| Sudan-AB1-2009 | Sudan | 2009 | Africa-3 | HQ378179 | HQ378187 | HQ378183 |
| Matin | Pakistan | 1976 | Asia-1 | AF527810 | AF467769 | AY422208 |
| TADJ/HU8966 | Tajikistan | 1990 | Asia-2 | AY049083 | AY179962 | AY720893 |
| C-68031 | China | 1968 | Asia-2 | DQ211642 | DQ211629 | DQ211616 |
| 79121 M18 | China | 2004 | Asia-2 | GU477494 | GU477493 | GU477492 |
| AP92 | Greece | 1975 | Europe-2 | DQ211638 | DQ211625 | DQ211612 |
| ArD39554 | Mauritania | 1984 | Africa-3 | DQ211641 | DQ211628 | DQ211615 |
| 30908 | Russia,  Astrakhan region | 2002 | Europe-1  (Va subgroup) | - | - | AY675240 |
| STV/HU29223 | Russia,  Stavropol territory | 2000 | Europe-1  (Va subgroup) | AF481802 | AF489586 | - |
| ROS/HUVLV-100 | Russia,  Rostov region | 2003 | Europe-1  (Vb subgroup) | DQ206447 | DQ206448 | AY995166 |
| ROS/TI28044 | Russia,  Rostov region | 2000 | Europe-1  (Vb subgroup) | AY277672 | - | - |
| Kashmanov | Russia,  Rostov region | 1967 | Europe-1  (Vb subgroup) | DQ211644 | DQ211631 | DQ211618 |
| VLG/TI29414 | Russia,  Volgograd region | 2000 | Europe-1  (Vb subgroup) | - | AY179961 | - |
| K229-243 | Russia,  Astrakhan region | 1984 | Europe-1  (Vc subgroup) | KX013467 | KX013466 | KX013465 |
| Kosova-Hoti | Kosovo | 2001 | Europe-1 | DQ133507 | EU037902 | EU044832 |
| Kosovo/9553/2001 | Kosovo | 2001 | Europe-1 |  | AY675511 |  |
| V46/13 | Bulgaria | 2013 | Europe-1 | KR011837 | KR092379 |  |
| Turkey200310849 | Turkey | 2003 | Europe-1 | DQ211649 | DQ211636 | DQ211623 |
| Turkey-Kelkit06 | Turkey | 2006 | Europe-1 | GQ337053 | GQ337054 | GQ337055 |
| Yozgat19-2012 | Turkey | 2012 | Europe-1 | KR092375 | - | - |
| Yozgat207-2011 | Turkey | 2011 | Europe-1 | KR092377 | - | - |
| Eskisekir 23-2012 | Turkey | 2012 | Europe-1 | KR092376 | - | - |
| Corum 1096-2016 | Turkey | 2016 | Europe-1 | KR092378 | - | - |
| Iran-Gilan69 | Iran | 2012 | Europe-1 | KJ027521 |  |  |
| 1-CRIMEA/HU-2015 | Russia,  Crimea Republic | 2015 | Europe-1  (Vd subgroup) | KU161586 | KU161584 | KU161582 |
| 1231-CRIMEA/TI-2015 | Russia,  Crimea Republic | 2015 | Europe-1  (Vd subgroup) | KU161587 | KU161585 | KU161583 |
| 1237-CRIMEA/TI-2015 | Russia,  Crimea Republic | 2015 | Europe-1  (Vd subgroup) | KU161581 | KU161579 | KU161577 |
| 1193-CRIMEA/TI-2015 | Russia,  Crimea Republic | 2015 | Europe-1  (Vd subgroup) | KU161580 | KU161578 | KU161576 |
