# Supplementary material for: Molecular epidemiology of Crimean-Congo hemorrhagic fever virus in Russia
Source: PLoS One. 2022 May 12;17(5):e0266177. doi: 10.1371/journal.pone.0266177 (PMC9098019; doi:10.1371/journal.pone.0266177)
Supplement: S2 File — The ages of the clades are shown at the node. Reassortant strains are marked. (DOC) [file pone.0266177.s005.doc]

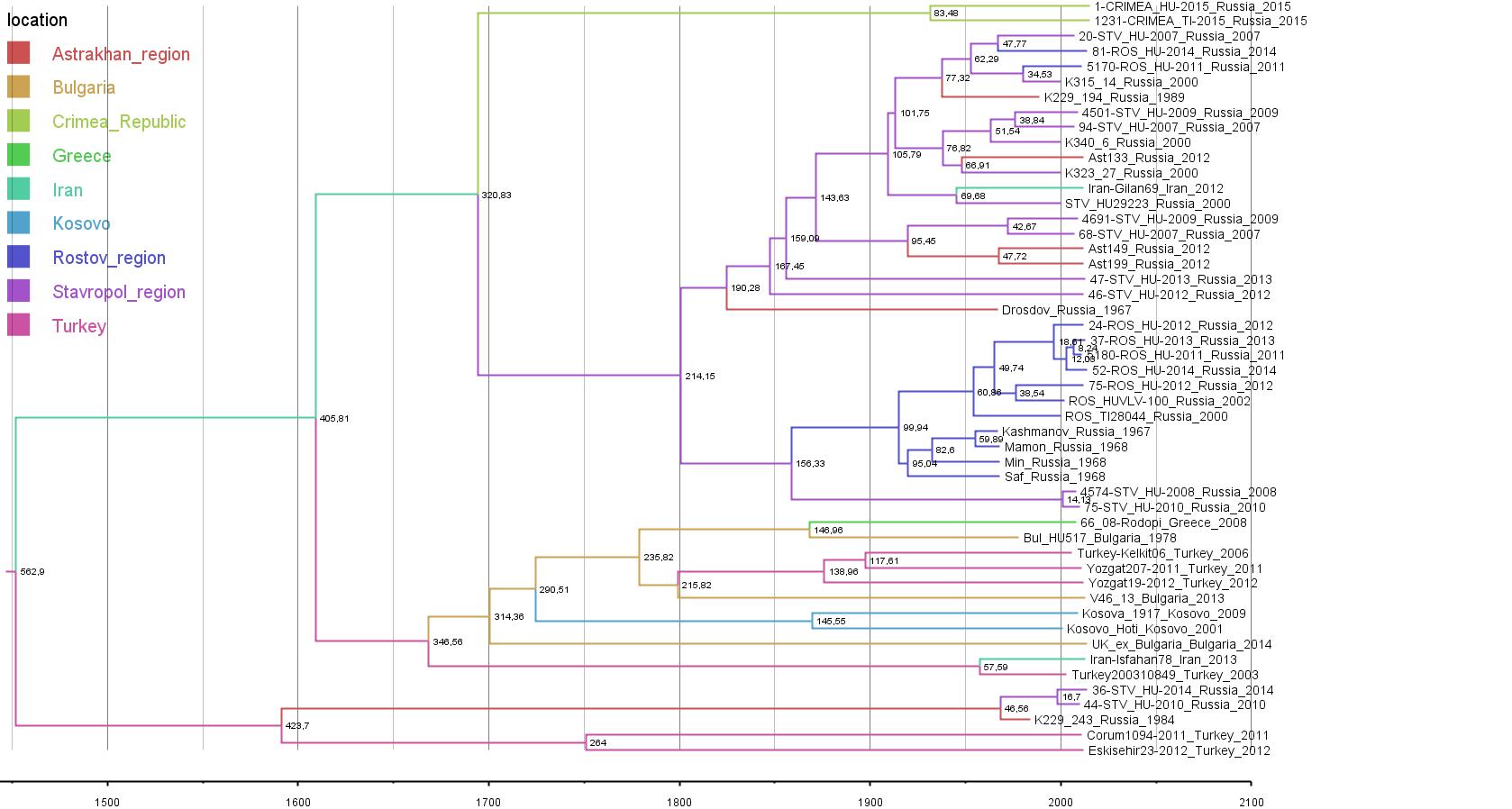


Va

Vb

Vd

Vc

MCC tree based on the complete ORF of the S-segment (the dataset includes 50 strains belonging to the Europe-1 genotype).

Discrete phylogeography reconstruction. The ages of the clades are shown at the nodes. Reassortant strains are marked.


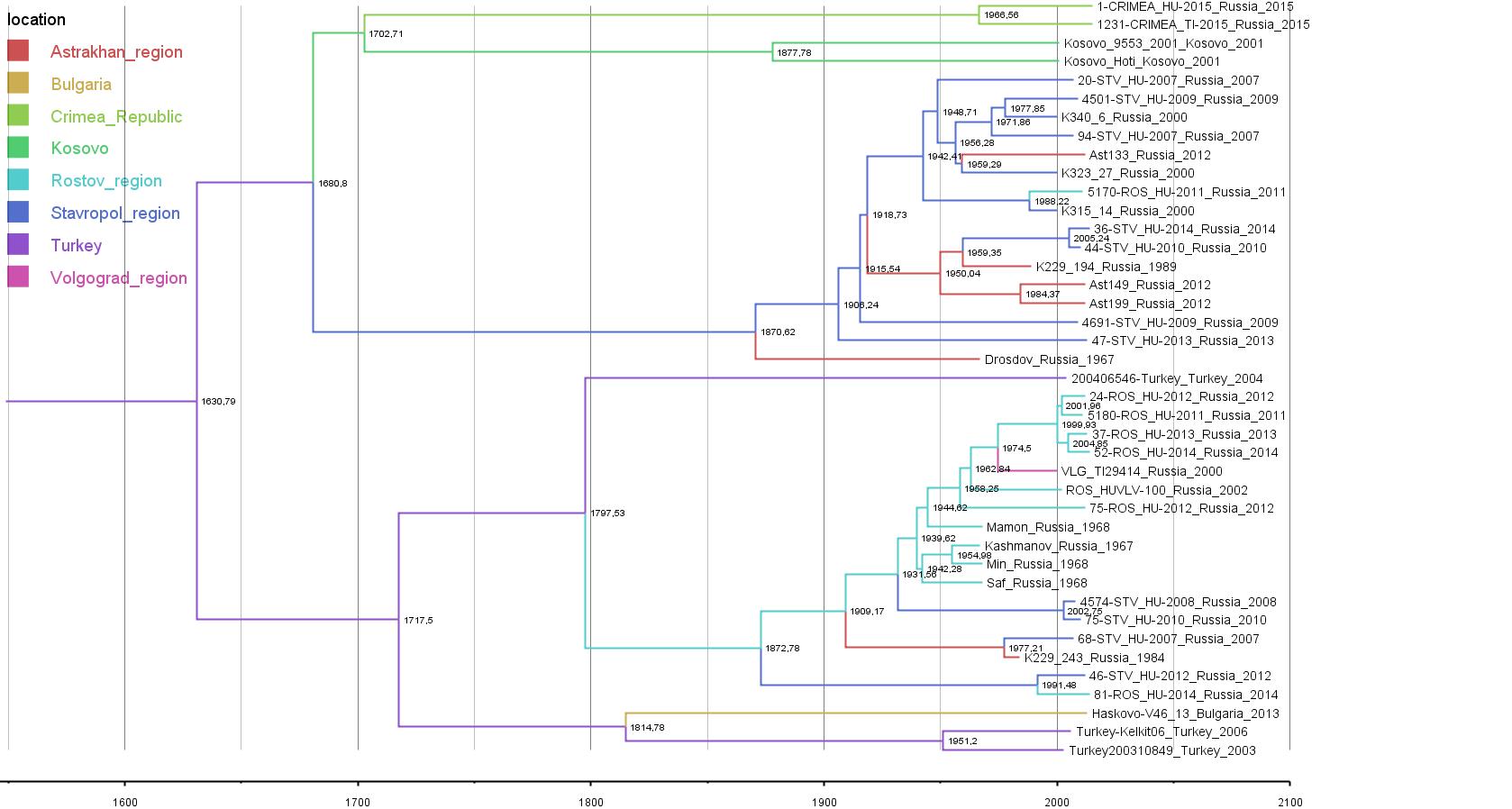


Va

Vb

Vd

MCC tree based on the complete ORF of the M-segment (the dataset includes 41 strains belonging to the Europe-1 genotype).

Discrete phylogeography reconstruction. The ages of the clades are shown at the nodes. Reassortant strains are marked.


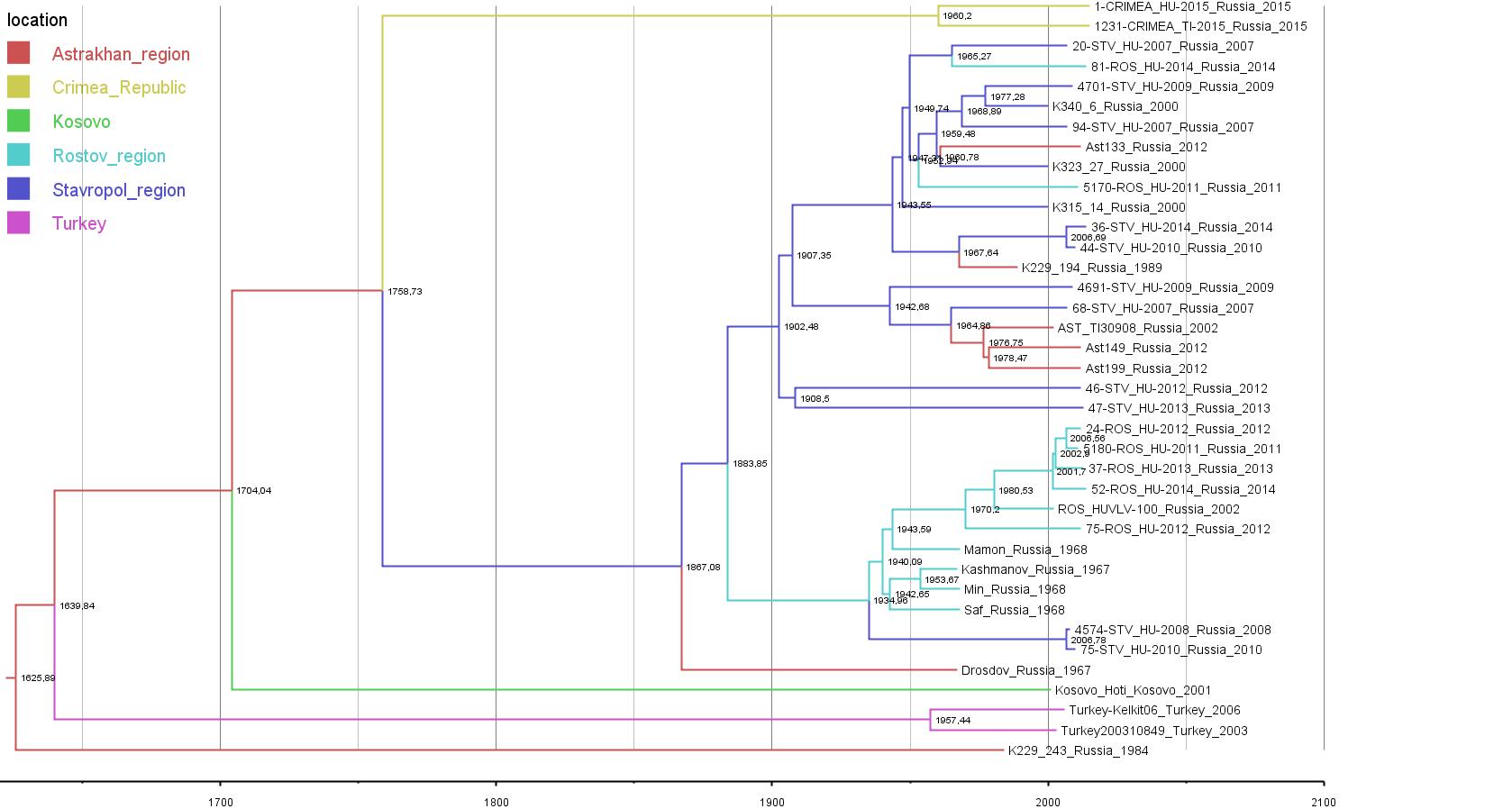


Va

Vb

Vd

Vc

MCC tree based on the complete ORF of the L-segment (the dataset includes 38 strains belonging to the Europe-1 genotype).

Discrete phylogeography reconstruction. The ages of the clades are shown at the nodes. Reassortant strains are marked.
